# Use of potentially driver-impairing drugs among older drivers

**DOI:** 10.1186/s12877-021-02726-5

**Published:** 2022-01-03

**Authors:** Sarah Zitoun, Edouard Baudouin, Emmanuelle Corruble, Jean-Sébastien Vidal, Laurent Becquemont, Emmanuelle Duron

**Affiliations:** 1 University of Paris-Saclay, Inserm, CESP, Team MOODS, 94270 Le Kremlin-Bicêtre, France; 2 Assistance Publique - Hôpitaux de Paris, Bicêtre Hospital, Department of Psychiatry, 94270 Le Kremlin-Bicêtre, France; 3 Assistance Publique - Hôpitaux de Paris, Paul Brousse Hospital, Department of Geriatric, 94800 Villejuif, France; 4 Assistance Publique - Hôpitaux de Paris, Hôpital Broca, F-75013 Paris, France; 5 Université de Paris, EA 4468, F-75013 Paris, France

**Keywords:** Driving, Potentially driver-impairing drugs, Older people, Primary care

## Abstract

**Background:**

Road safety is a major issue among seniors. Potentially Driver-Impairing (PDI) drugs are known to increase the risk of car accident. The aim of this cross-sectional study was to describe PDI-drug consumption among older drivers and determine associated factors.

**Methods:**

The S.AGES cohort is a French non-interventional real-life prospective study of 3700 community-dwelling participants aged ≥65 years old, suffering from type 2 diabetes (T2DM), chronic pain or atrial fibrillation (AF). Baseline data of drivers with known treatment (*n* = 1783) were used for the analyses. PDI drugs were defined according to the French classification.

**Results:**

One thousand seven hundred eighty-three drivers were included (66% males; mean age 76 (Standard deviation = 5.78) years old). 21% (*n* = 373) took PDI drugs, 64% of which took only one (*n* = 239). The most frequent PDI drugs were: Zolpidem (11%; *n* = 60); Zopiclone (8%; *n* = 45); Bromazepam (8%; *n* = 44); Tramadol (7%; *n* = 39); Pregabalin (6%; *n* = 31). Drivers taking PDI drugs had more often chronic pain (OR [95% CI] = 2.30 [1.54–3.46]), history of depressive disorder (4.28 [3.00–6.14]) and polypharmacy (taking at least 5 different medications; 4.32 [2.97–6.41]), and less often T2DM (0.54 [0.37–0.79]), and AF (0.48 [0.32–0.71]). Conversely, they had a lower Activities of Daily Living score (0.34 [0.17–0.68]).

**Conclusions:**

The rate of aged drivers in the S.AGES cohort taking PDI drugs is concerning and highlights the need to carefully assess and reassess PDI-drug prescriptions in this population, particularly hypnotics, anxiolytics and opioids.

**Trial registration:**

ClinicalTrials.gov NCT01065909 (First posted: February 9th, 2010).

**Supplementary Information:**

The online version contains supplementary material available at 10.1186/s12877-021-02726-5.

## Background

Worldwide population aged 65 and older will double in 2050: it will reach 1.5 billion by then. In Europe and Northern America, a 48% increase is projected. Moreover, the worldwide number of subjects aged 80 and older will triple to reach 426 million in 2050 [1].

Driving among older people is a matter of great concern. According to the Insurance Institute for Highway Safety, the proportion of the population ≥ 70 years old with driving licenses in United States (US) grew from 73% in 1997 to 83% in 2018 [2]. In European Union (EU) by 2030, a quarter of licensed drivers will be aged 65 and older [3]. Furthermore, even though older driver involvement rate in traffic accident globally declined these last couple of years [4, 5], older drivers remain significantly implicated in car accidents. In 2017, in the US, the number of fatal car accident per million miles traveled was 2.1 for drivers aged 75–79 and reached 7.6 for drivers aged 85 and older [2]. In 2013, in EU, 20% of all deaths among car drivers involved older people [3].

Aging is associated with altered physiological functions and diseases that can impair ability to drive, like visual impairments, musculoskeletal disorders, diabetes, dementia [6], depression [6, 7], and use of psychotropic drugs [7, 8]. Potentially modifiable factors that could alleviate the risk of car accident in older people are of interest. In this way, Potentially Driver-Impairing (PDI) drugs consumption among older drivers ought to be studied. To date, real life data on driving in older people are scarce in France, particularly concerning the use of PDI drugs.

The objective of this study was to describe the use of PDI drugs among older drivers of the S.AGES cohort, and to analyze factors associated with their consumption.

## Methods

### Population

The S.AGES study is a French non-interventional real-life prospective study that included participants from April 2009 to June 2011. Characteristics of the cohort have already been published [9]. A total of 3700 community-dwelling participants aged 65 and older have been included by 760 French general practitioners (GP). The cohort was composed of 3 sub-cohorts: participants suffering from type 2 diabetes (T2DM), chronic pain or atrial fibrillation (AF). Investigators were randomized into one of the 3 sub-cohorts and could only include participants in the sub-cohort they had been randomized to. Each investigator included 1/3 of participants aged 65–75 years old, and 2/3 of participants aged 75 and older. Participants were assessed by their GP every 6 months for 3 years. The study was approved by the Ethics committee (Comité de protection des personnes Ile de France XI) on January 15, 2009 (ref 09006) and by the French National Agency for Medicines and Health Products (ANSM) on February 6, 2009 (ref B81333–40) (ClinicalTrials.gov NCT01065909).

The inclusion criteria were: community-dwelling adult aged 65 and older; resident in metropolitan France; covered by health care insurance; who signed the informed consent form and affected by one of the 3 following conditions: confirmed AF within 12 months before inclusion; pain for more than 3 months and requiring care; T2DM treated at inclusion by an oral and/or injectable anti-diabetic medication.

The exclusion criteria were: nursing home resident at the time of inclusion; unable to understand the goal of the study and to give informed consent; impossible follow-up after inclusion (moving planned, homeless); already included in another therapeutic trial; non-cardiovascular disease with less than 3-month life expectancy; transient AF (related to thyrotoxicosis, excessive alcohol consumption, myocarditis, pericarditis, acute phase of myocardial infarction, pulmonary embolism, metabolic disorders, electrocution) and AF following heart surgery within 3 months before inclusion.

Socio-demographic, clinical and treatment data were recorded at inclusion, and updated at each visit except for driving status.

The socio-demographic data were: age, sex, smoking status (never, former, current), alcohol consumption (daily alcohol consumer or not), living environment (rural (or semi-rural) or urban area), living arrangement (at home alone or with spouse, or in an assisted living facility), driving status (active driver or not, according to participants’ answer at the yes/no question “Do you drive a motorized vehicle?”, with no detail required about frequency of driving nor having a driver’s license) at inclusion, educational level (achievement ≥9th grade or less), work history, disability assessed by the Activities of Daily Living scale (ADL, maximum score 6 indicating no disability in activities of daily living) [10] and the Instrumental Activities of Daily Living scale (IADL, maximum score 4 indicating no instrumental disability) [11], financial or logistical supports, and paramedical assistance (home nursing care, pedicure or physiotherapy).

The clinical data recorded were: participant’s apparent age (younger than, same as, or older than participant’s chronological age) according to GP’s gut feeling, body mass index (BMI, kg/m^2^), estimated glomerular filtration rate (eGFR in mL/min/1.73m^2^, calculated by the Modification of Diet in Renal Disease formula) [12], cardiovascular diseases (arterial disease, AF, high blood pressure, heart failure with severity assessed by the New York Heart Association (NYHA) classification), neurological diseases (Parkinson’s disease, history of severe stroke, cognition assessed by the Mini-Mental State Examination (MMSE, maximum score 30 indicating no cognitive impairment) [13], psychiatric disorders (probable clinical depression assessed by a score ≥ 10 at the 15-item Geriatric Depression Scale (GDS-15, maximum score 15, the higher, the more important risk of depression) [14], history of depressive disorder), and several other medical conditions: thyroid dysfunction, T2DM, history of severe hypoglycemia (within less 12 months before inclusion), respiratory diseases (sleep apnea, chronic obstructive pulmonary disease (COPD) or pulmonary fibrosis), arthropathy (symptomatic osteoarthritis or chronic inflammatory rheumatic disease), history of falls (within 12 months before inclusion) and chronic pain.

The original study provided precise information about medications prescribed at baseline: name (International Nonproprietary (INN) name or trade one), Anatomical Therapeutic Chemical Classification System (ATC) code, dosage, therapeutic indication, number of unity per intake, number of intake per day, start and end dates of prescription [9]. In this study, treatment data recorded were the use of at least one PDI drug and polypharmacy (defined here as taking at least 5 different medications) [15]. Based on the ATC codes, each molecule was classified as PDI drug according to the French classification that distinguishes 3 levels of warning:

- level 1: low risk at driving; the drug intake does not contraindicate driving, but drivers are recommended to read the drug notice;
- level 2: pharmacodynamic effects of the drug predominates over individual susceptibility; the drug intake may alter driving ability; driving requires the advice of a health professional;
- and level 3: pharmacodynamic effects of the drug highly contraindicate driving; driving again requires the advice of a medical practitioner [16] (see Additional file 1).

In this study, a drug was classified as “PDI drug” if it was categorized at level 2 or 3 of the French classification, as level 1 does not contraindicate driving.

**Fig. 1:**
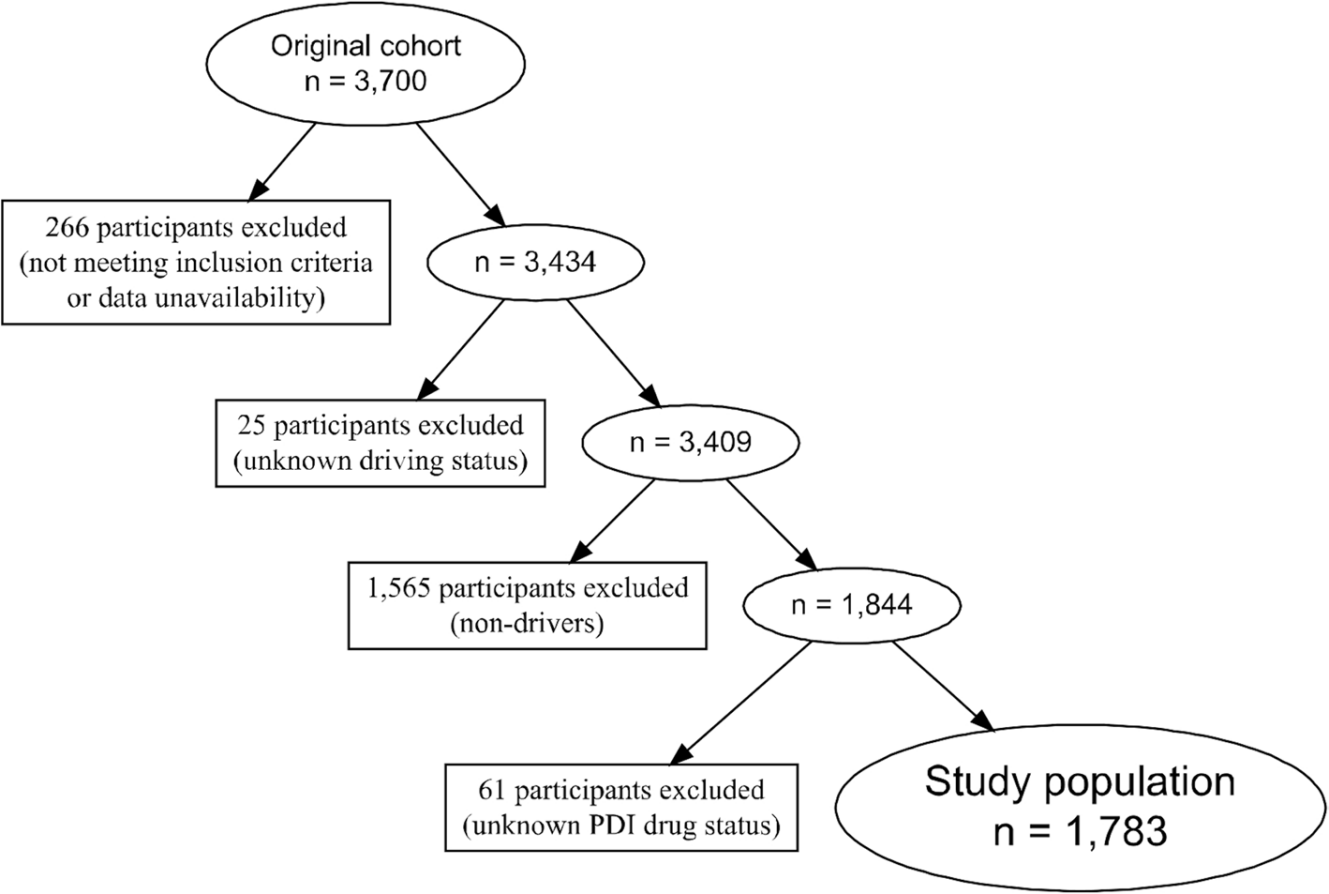
Flowchart of the study population. Abbreviation. PDI, Potentially Driver-Impairing

Among the 3700 participants included in the original cohort, 266 were excluded because they did not meet inclusion criteria or because of data unavailability [9]. Among the 3434 remaining participants, 25 were excluded because of unknown driving status. Among the 3409 remaining participants, 1565 were excluded because they were non-drivers. Among the 1844 drivers, 61 were excluded because of unknown PDI status (having at least one PDI drug in their treatment or not). Only drivers with information on their PDI status at baseline (*n* = 1783) were included in this cross-sectional analysis of the S.AGES study (Fig. 1). Drivers were categorized according to their use of PDI drugs: drivers taking at least one PDI drug (PDI+); drivers without any PDI drug in their treatment (PDI-).

### Statistics

All statistical analyses were performed with the R Software (version 4.0.3; R Core Team (2019). R: A language and environment for statistical computing. R Foundation for Statistical Computing, Vienna, Austria. URL https://www.R-project.org/). The descriptive parameters for the qualitative variables are presented as counts and percentages. The descriptive parameters for the quantitative variables are presented as means and standard deviations (M (SD)). Normal distribution hypotheses were tested using Shapiro-Wilk tests. The relationships between the dependent variable (PDI status) and the participants’ characteristics were first tested using Chi-squared test or Fisher’s exact test for categorical data, and Student’s t-test or non-parametric Mann-Whitney test for continuous data. Quantitative variables with missing values accounting for 10% or more of the sample were not included in the analyses. All variables with a *p*-value < 0.10 in univariate analysis were selected for the multivariate one, after a pre-selection on their correlation. Then a logistic regression model was built with PDI status as dependent variable by a stepwise selection of the independent variables on complete observations. The final model was adjusted for all clinically relevant variables from the literature, and for T2DM, AF and chronic pain in order to take into account the possible selection bias induced by sub-cohorts. The adjusted odds ratios (OR) were calculated with their 95% confidence intervals (95% CI). Two-sided *p*-values < 0.05 were considered statistically significant.

## Results

**Fig. 2:**
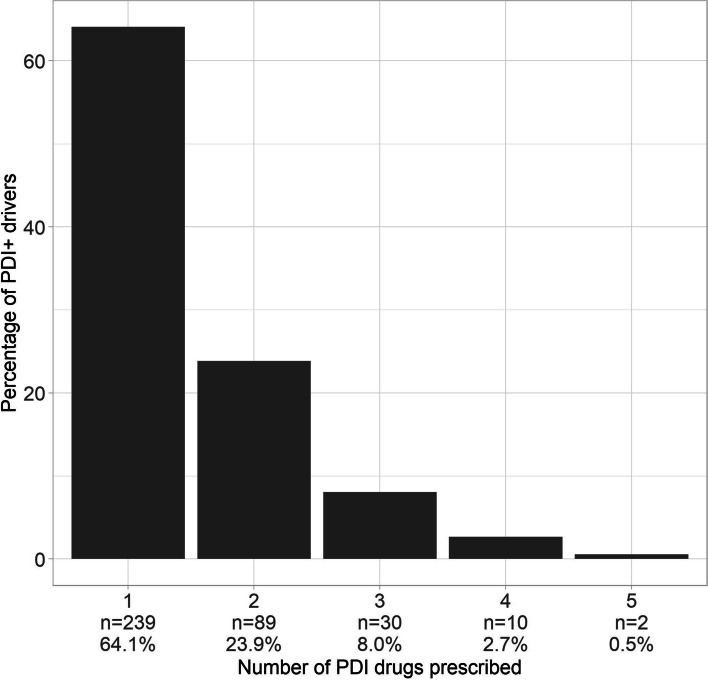
Rates of PDI+ drivers by number of PDI drugs prescribed. Abbreviations. PDI, Potentially Driver-Impairing; PDI+ drivers, Drivers taking at least one PDI drug

In this S.AGES study, 1783 drivers had information on their PDI-drug consumption at baseline. Their characteristics at baseline are detailed in Table 1, as a whole and according to their PDI status. A third of the drivers were included in the chronic pain sub-cohort (*n* = 595), a third in the AF sub-cohort (*n* = 603), and a last third in the T2DM sub-cohort (*n* = 585). Mean age was 75.9 (5.8) years old, 65.5% (*n* = 1168) were males and 20.9% (*n* = 373) were PDI+. Most of the PDI+ drivers took only one PDI drug (64.1%; *n* = 239) (Fig. 2). The most frequently prescribed PDI drugs were: Zolpidem (10.7%; n = 60); Zopiclone (8%; *n* = 45); Bromazepam (7.8%; *n* = 44); Tramadol (6.9%; *n* = 39); Pregabalin (5.5%; *n* = 31).

**Table 1:** Descriptive and univariate analyses of factors associated with PDI-drug consumption in drivers

General characteristics, % (n)	Missing, % (n)	All	PDI-	PDI+	p^**a**^
		n = 1783	***n*** = 1410	***n*** = 373	
**Sub-cohorts**	0 (0)				**< 0.001**
**Chronic pain**		33.37 (595)	28.37 (400)	52.28 (195)	
**Atrial fibrillation**		33.82 (603)	36.88 (520)	22.25 (83)	
**Type 2 diabetes**		32.81 (585)	34.75 (490)	25.47 (95)	
**Males**	0 (0)	65.51 (1168)	68.94 (972)	52.55 (196)	**< 0.001**
**Age, M (SD)**	0 (0)	75.88 (5.78)	75.98 (5.74)	75.52 (5.93)	0.254
**Apparent age**	0.28 (5)				**0.010**
**< Chronological age**		23.84 (425)	24.89 (351)	19.84 (74)	
**= Chronological age**		68.93 (1229)	68.65 (968)	69.97 (261)	
**> Chronological age**		6.95 (124)	6.17 (87)	9.92 (37)	
**BMI, M (SD)**	3.48 (62)	28.18 (4.76)	28.17 (4.67)	28.22 (5.09)	0.793
**eGFR, M (SD)**	0.18 (328)	73.64 (20.43)	73.83 (20.17)	72.91 (21.39)	0.472
**Smoking status**	0.45 (8)				0.185
**Never**		63.15 (1126)	62.34 (879)	66.22 (247)	
**Former**		32.25 (575)	33.26 (469)	28.42 (106)	
**Current**		4.15 (74)	3.97 (56)	4.83 (18)	
**Alcohol consumption**	1.12 (20)	36.34 (648)	37.73 (532)	31.10 (116)	0.019
**Living area**	0 (0)				0.653
**Rural or semi-rural**		56.25 (1003)	56.52 (797)	55.23 (206)	
**Urban**		43.75 (780)	43.48 (613)	44.77 (167)	
**Social lifestyle**	0 (0)				**< 0.001**
**Alone at home**		25.86 (461)	22.77 (321)	37.53 (140)	
**Accompanied at home**		73.75 (1315)	76.81 (1083)	62.20 (232)	
**Assisted living facility**		0.39 (7)	0.43 (6)	0.27 (1)	
**Achievement ≥ 9th Grade**	1.51 (27)	63.99 (1141)	64.47 (909)	62.20 (232)	0.524
**Work history**	0.62 (11)	90.91 (1621)	91.77 (1294)	87.67 (327)	**0.010**
**ADL score, M (SD)**	0.34 (6)	5.94 (0.25)	5.95 (0.19)	5.87 (0.38)	**< 0.001**
**IADL score, M (SD)**	0.28 (5)	3.91 (0.47)	3.92 (0.44)	3.86 (0.54)	**0.002**
**Financial or logistical supports**	0.17 (3)	90.24 (1609)	90.28 (1273)	90.08 (336)	0.958
**Paramedical assistance**	1.18 (21)	36.68 (654)	33.69 (475)	47.99 (179)	**< 0.001**
**Arterial disease**	0.39 (7)	19.07 (340)	18.65 (263)	20.64 (77)	0.360
**Atrial fibrillation**	0.28 (5)	39.37 (702)	42.34 (597)	28.15 (105)	**< 0.001**
**High blood pressure**	0.11 (2)	77.96 (1390)	78.87 (1112)	74.53 (278)	0.065
**Heart failure**	1.18 (21)				0.202
**No**		88.67 (1581)	88.51 (1248)	89.28 (333)	
*Mild to moderate heart failure,* **NYHA I-II**		7.40 (132)	7.80 (110)	5.90 (22)	
*Severe heart failure,* **NYHA III-IV**		2.75 (49)	2.48 (35)	3.75 (14)	
**MMSE score**	15.14 (270)				**0.040**
*Probable moderate to severe cognitive impairment,* **< 24**		4.88 (87)	4.47 (63)	6.43 (24)	
*Probable mild cognitive impairment,* **[24–27]**		14.47 (258)	13.55 (191)	17.96 (67)	
*No cognitive impairment,* **≥ 27**		65.51 (1168)	66.24 (934)	62.73 (234)	
**Parkinson’s disease**	0.39 (7)	0.73 (13)	0.21 (3)	2.68 (10)	**< 0.001**
**History of severe stroke**	0.45 (8)	2.02 (36)	1.84 (26)	2.68 (10)	0.310
**Probable clinical depression** ***(GDS-15 score ≥ 10)***	22.27 (397)	4.99 (89)	4.33 (61)	7.51 (28)	**0.007**
**History of depressive disorder**	0.06 (1)	17.33 (309)	10.92 (154)	41.55 (155)	**< 0.001**
**Thyroid dysfunction**	1.29 (23)	10.54 (188)	9.57 (135)	14.21 (53)	**0.011**
**Type 2 diabetes**	0.28 (5)	43.02 (767)	45.18 (637)	34.85 (130)	**< 0.001**
**History of severe hypoglycaemia**	1.35 (24)	0.62 (11)	0.50 (7)	1.07 (4)	0.259
**Sleep apnea**	0.39 (7)	4.82 (86)	4.61 (65)	5.63 (21)	0.417
**Pulmonary chronic obstructive disease or fibrosis**	0.39 (7)	8.69 (155)	8.23 (116)	10.46 (39)	0.177
**History of fall**	0.73 (13)	6.62 (118)	5.25 (74)	11.80 (44)	**< 0.001**
**Arthropathy**	0.22 (4)	46.83 (835)	42.62 (601)	62.73 (234)	**< 0.001**
**Chronic pain**	0.17 (3)	54.23 (967)	48.51 (684)	75.87 (283)	**< 0.001**
**Polypharmacy**	0 (0)	57.82 (1031)	52.06 (734)	79.62 (297)	**< 0.001**

*Abbreviations. M (SD)* mean (standard deviation), *BMI* body mass index in kg/m^2^, *eGFR* glomerular filtration rate in mL/min/1.73m^2^, *ADL score* Activities of Daily Living score (maximum score 6 indicating no disability in activities of daily living), *IADL score* Instrumental Activities of Daily Living score (maximum score 4 indicating no instrumental disability), *NYHA* New York Heart Association, *MMSE score* Mini-Mental State Examination score (maximum score 30 indicating no cognitive impairment), *GDS-15 score* 15-item Geriatric Depression Scale score (maximum score 15, the higher, the more important risk of depression), *PDI* Potentially Driver-Impairing, *PDI+ drivers* Drivers taking at least one PDI drug, *PDI- drivers* Drivers without any PDI drug in their treatment

^a^ Chi-squared or Fisher’s exact test for categorical data, Student’s t-test for continuous data and Mann-Whitney test for non-parametric continuous data. A *p*-value < 0.05 is significant

**Fig. 3:**
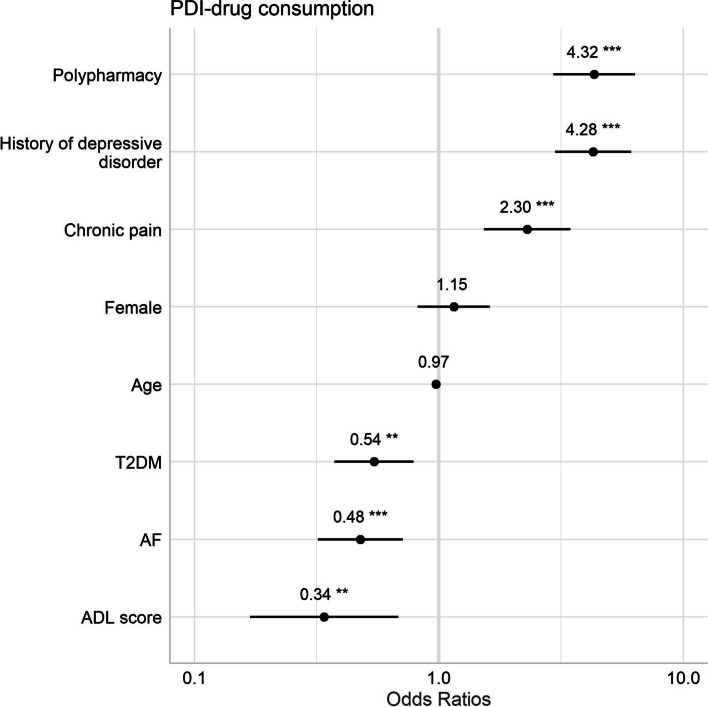
Multivariate analysis of factors associated with PDI-drug consumption in drivers. Abbreviations. PDI, Potentially Driver-Impairing; T2DM, Type 2 diabetes; AF, Atrial fibrillation; ADL score, Activities of Daily Living score (maximum score 6 indicating no disability in activities of daily living). *, *p* <  0.05; **, *p* <  0.01; ***, *p* <  0.001. The model was adjusted for age, sex, history of depressive disorder, polypharmacy, ADL score, and the 3 diseases relating to the sub-cohorts (T2DM, AF and chronic pain)

In multivariate analysis, the final model was adjusted for age, sex, history of depressive disorder, polypharmacy, ADL score, T2DM, AF and chronic pain. PDI+ drivers had more often chronic pain (OR [95% CI] = 2.30 [1.54–3.46]), history of depressive disorder (4.28 [3.00–6.14]) and polypharmacy (4.32 [2.97–6.41]), and less often T2DM (0.54 [0.37–0.79]), and AF (0.48 [0.32–0.71]). Moreover, PDI+ drivers had a lower Activities of Daily Living score (0.34 [0.17–0.68]) (Table 2 and Fig. 3).

**Table 2:** Multivariate analysis of factors associated with PDI-drug consumption in drivers

	OR	95% CI	p-value
ADL Score	**0.34**	**0.17–0.68**	**0.002**
Atrial fibrillation	**0.48**	**0.32–0.71**	**< 0.001**
Type 2 diabetes	**0.54**	**0.37–0.79**	**0.001**
Age	0.97	0.95–1.00	0.066
Female	1.15	0.82–1.62	0.418
Chronic pain	**2.30**	**1.54–3.46**	**< 0.001**
History of depressive disorder	**4.28**	**3.00–6.14**	**< 0.001**
Polypharmacy	**4.32**	**2.97–6.41**	**< 0.001**

The model was adjusted for age, sex, history of depressive disorder, polypharmacy, ADL score, and the 3 diseases relating to the sub-cohorts (type 2 diabetes, atrial fibrillation and chronic pain)

*Abbreviations. PDI* Potentially Driver-Impairing, *OR* Odds Ratio, *95% CI* 95% Confidence Interval, *ADL score* Activities of Daily Living score (maximum score 6 indicating no disability in activities of daily living)

## Discussion

In this study, more than 20% of drivers were treated with at least one PDI drug (*n* = 373). The most frequent PDI drugs prescribed to drivers were Z-drug hypnotics (Zolpidem and Zopiclone), one anxiolytic (Bromazepam) and analgesics (Tramadol and Pregabalin). Compared to drivers without any PDI drug in their treatment, PDI+ drivers had more disability and more often a history of depressive disorder and were receiving more often polypharmacy. They had more frequently chronic pain and less frequently T2DM and AF.

The percentage of PDI+ drivers in this study was lower than in previous research. A study conducted in U.S. among 2990 older drivers aged 65–79 years old found that 70% were under central nervous system agent [17]. Another one found 68.9% of PDI drug consumers among 225 drivers (mean age: 68 (12.8) years old) [18], according to the PDI medication list defined by Leroy and Morse in 2008, which includes not only central nervous system agents but also numerous other system agents (hematologic, cardiovascular-renal, gastrointestinal, metabolic, hormonal, neurologic, ophthalmic, otologic, antiparasitic, respiratory and analgesic ones) [19]. These discrepancies may be explained by the fact that not all central nervous system agents were taken into account in the present study, but only those impairing driving performance according to the French classification [16] (see Additional file 1). Furthermore, the studies of Hetland et al. and Hill et al. included medically impaired drivers referred to an occupational therapy-based driving evaluation clinic inflating in all likelihood the rate of PDI-drug consumption [17, 18]. Also, self-medication was not assessed in this study which can have minimized the prevalence of PDI-drug consumption, whereas self-medication is frequent in the older population [20], especially analgesics [20], and in France, benzodiazepines [21], both of them being PDI drugs.

In this study, the most frequent PDI drugs were Z-drug hypnotics (Zolpidem (10.7%) and Zopiclone (8%)), followed by one benzodiazepine (Bromazepam (7.8%), and analgesics (Tramadol (6.9%) and Pregabalin (5.5%)). These results are consistent with previous research. One study found that Zopiclone and Diazepam were the two most frequent single legal drugs found in blood samples of older drivers suspected of driving under the influence of drugs. In this study, ethanol was detected in 81% of blood samples, Zopiclone in 9.8% and Diazepam in 9.3% [22]. In the study of Hetland et al. in 2014, benzodiazepines, opioids and non-benzodiazepine hypnotics accounted respectively for 6.6, 4.5 and 4% of PDI drugs used among the 225 drivers [18]. Benzodiazepines are overprescribed in older people [21, 23], particularly in the treatment of insomnia and anxiety disorders, which are both frequent in older adults [24, 25]. Non-benzodiazepine hypnotics, known as Z-drugs (Zopiclone and Zolpidem for example) are also commonly used to treat insomnia because they are often perceived as safer than benzodiazepines, while they are associated with an increased risk of falls and fractures [26]. Moreover, older subjects frequently suffer from chronic pain [27, 28], which can explain the high rates of opioid use in this population (6–9%) [27, 29]. Opioid misuse is also frequent in older people (1–3%) [29], perhaps because of mood modifying effects of opioid agents, which are known to increase serotonin levels and cause addiction [30].

Our study found several factors associated with PDI-drug consumption. First, taking at least one PDI drug was associated with polypharmacy, which is frequently observed in older people [31–33]. Second, PDI-drug consumption was associated with history of depressive disorder and chronic pain, which is consistent with the 5 most frequent PDI drugs prescribed in this study. Indeed, insomnia and anxiety are frequently observed in depressed older people [34], and also described as a risk factor for late life depression [35]. Moreover, previous research found that chronic pain is a risk factor for depression in older adults, and similarly, that depressed older people were more likely to suffer from chronic pain, corroborating the hypothesis, that neuroinflammation could be a common pathogenic factor of chronic pain and depression [36]. Surprisingly, no antidepressant was found in the 5 most frequent PDI drugs, which may be explained by the fact that only 7.5% of PDI+ drivers had probable clinical depression (according to their GDS-15 score at inclusion), suggesting that insomnia, anxiety and chronic pain would be residual or prodromal symptoms of depression in these patients. Conversely, PDI+ drivers in this study suffered less frequently from T2DM and AF. This may be explained by a better management of patients suffering from these chronic diseases, with regular revision of prescriptions. Finally, PDI+ drivers had more disability than PDI- ones, which might be driven by the association of symptoms treated with PDI medications (like insomnia, anxiety or chronic pain) and medical comorbidity [24, 27, 37].

These results are a matter of great concern since the increased risk of car accident in drivers taking PDI drugs is widely documented [8, 18, 38, 39], especially for benzodiazepines [8, 40], Z-drug hypnotics [8, 41, 42] and opioids [8, 39, 43, 44]. Furthermore, most of the factors found here associated with PDI-drug consumption in older drivers are also associated with a higher risk of car accident, in particular depression [6, 7], chronic pain known to alter performance on attention tasks [45], and polypharmacy [17, 18]. In the study of Hetland et al., the average medically impaired driver was taking 5.9 (3.7) total routine medications [18], while half of older drivers interviewed for the AAA Foundation for Traffic Safety study were taking seven or more medications [17]. Furthermore, PDI drugs tolerance is more likely to be poorer in older subjects than younger ones because of pharmacokinetic changes associated with ageing, like altered hepatic and renal functions that increase plasma elimination half-life, leading to increase of drug side effects like impaired attention, increased reaction time, hypersomnolence and confusion [46].

The concerning rate of aged drivers taking PDI drug might raise the question of searching for ways to decrease it. If no recommendation can be formally given to GPs based on this study because of several limitations, some aspects of drug prescription in aged population should be noted. First, the results of this study show the importance of questioning patients about their driving habits and considering them before prescribing. They might also highlight the importance of informing the patients and their families about the high risk of car accident while driving under the influence of PDI drugs, either prescribed or used for self-medication. Second, these results should also call for strategies to reduce the prescription of PDI drugs in older drivers and even avoid them when possible, keeping in mind that benzodiazepine and Z-drug hypnotic withdrawal can be achieved in the older population [47]. Whenever possible, alternative therapies should be considered: cognitive behavioral therapy is known to efficiently treat chronic pain [27], depression [36], anxiety [25] and insomnia (with stimulus control, sleep restriction, sleep hygiene and relaxation) [24], also acupuncture, hypnotherapy, physical exercise and relaxation have demonstrated some efficacy in the treatment of chronic pain and depression [36]. Third, since reducing polypharmacy should always be targeted, prescriptions should be regularly revised with the use of geriatrics tools like the Screening Tool of Older Person’s Prescriptions (STOPP) [48].

This study has some limitations. First, the results of this study have to be interpreted within the limit of its design. The fact that participants of the S.AGES cohort were included according to three specific diseases (T2DM, AF and chronic pain) might induce an inclusion bias, even though these diseases are highly prevalent in the older population [27, 49, 50]. Besides, Tramadol prescription in this study was probably higher than in the whole French population because one third of the participants were recruited in the chronic pain sub-cohort. Moreover, this study has a cross-sectional design: PDI status of participants may have changed during the follow-up. Another limitation is that the inclusion of the S.AGES data occurred about 10 years ago. The French list of PDI drugs has evolved during the last decade, which may have led to misestimate the number and distribution of such drugs among drivers. Nevertheless, according to the ANSM, in the whole French population in 2015, the rate of subjects taking benzodiazepines was still high (13.4% of the French population was prescribed a benzodiazepine at least once) [51]. Regarding the use of opioids, Tramadol consumption (alone or in association) has highly increased (+ 68%) between 2006 and 2017 [52]. Nevertheless, these most recent analyses of benzodiazepine (2015) and opioid analgesic (2017) consumptions by the ANSM were calculated from the whole French general population whatever the age, the driving status, and the state of health. They are therefore not easily comparable to descriptive analyses of this study population, which only comprises aged drivers with specific diseases.

Nevertheless, this study has several strengths: it was conducted in real life condition, with a large sample size, and data were recorded by patients’ GPs, with extensive socio-demographical, clinical and therapeutic information which gave a precise and detailed description of older drivers’ use of PDI drugs.

## Conclusions

This real life study showed a concerning rate of older drivers taking at least one PDI drug. These drivers, in comparison with those not taking any PDI drug, had more disability, more often a history of depressive disorder and were receiving more often polypharmacy. They had more frequently chronic pain and less frequently T2DM and AF. These observations highlight the huge importance for prescribers to frequently revise the relevance of prescribing PDI drugs in older drivers. They also highlight the need to assess driving risks, educate patients, and propose alternatives to maintain independence when driving cessation is raised. GPs are on the frontline to implement such measures, since they are first line health care providers in the older population. General or local authorities may also have a key role in providing alternative solutions to driving especially in rural areas.

## Supplementary Information

**Additional file 1.** Potentially driver-impairing drugs, categorized in level 2 or 3 of the French classification. Table summarizing potentially driver-impairing drugs categorized in level 2 or 3 of the French classification.

**Additional file 2.** Number of PDI drugs categorized at level 2 or 3 in the French classification, recorded in the original study. Table summarizing the number of potentially driver-impairing drugs categorized at level 2 or 3 in the French classification, recorded in the original study.

**Additional file 3.** Multivariate analyses of factors associated with PDI-drug consumption in drivers, respectively in the 3 sub-cohorts. Table summarizing the multivariate analyses of factors associated with potentially driver-impairing drug consumption in drivers, respectively in the 3 sub-cohorts (chronic pain, atrial fibrillation and type 2 diabetes).

## Data Availability

The datasets used and/or analysed during the current study are available from the corresponding author on reasonable request.

## Abbreviations

US: United States
EU: European Union
PDI: Potentially driver-impairing
GP: General practitioner
T2DM: Type 2 diabetes mellitus
AF: Atrial fibrillation
ANSM: French National Agency for Medicines and Health Products
ADL: Activities of Daily Living
IADL: Instrumental Activities of Daily Living
BMI: Body mass index
eGFR: Glomerular filtration rate
NYHA: New-York Heart Association
MMSE: Mini-mental State Examination
GDS-15: 15-item Geriatric Depression Scale
COPD: Chronic obstructive pulmonary disease
INN: International Nonproprietary
ATC: Anatomical Therapeutic Chemical Classification System
PDI +: Drivers taking at least one PDI drug
PDI-: Drivers without any PDI drug in their treatment
M (SD): Mean (Standard deviation)
OR (95% CI): Odds Ratio (95% confidence interval)
