# Supplementary material for: Use of potentially driver-impairing drugs among older drivers
Source: BMC Geriatr. 2022 Jan 3;22:4. doi: 10.1186/s12877-021-02726-5 (PMC8722131; doi:10.1186/s12877-021-02726-5)
Supplement: Supplementary file 1 — Additional file 1. Potentially driver-impairing drugs, categorized in level 2 or 3 of the French classification. Table summarizing potentially driver-impairing drugs categorized in level 2 or 3 of the French classification. [file 12877_2021_2726_MOESM1_ESM.pdf]

Additional file 1. Potentially driver-impairing drugs, categorized in level 2 or 3 of the French classification [1]

N01 - ANAESTHETICS

N01A - GENERAL ANAESTHETICS

**N01AB : Halogenated hydrocarbons**	
N01AB01	Halothane
N01AB04	Enflurane
N01AB06	Isoflurane
N01AB07	Desflurane
N01AB08	Sevoflurane

**N01AF : Non associated barbiturates**	
N01AF03	Thiopental

**N01AH : Opioid anaesthetics**	
N01AH01	Fentanyl
N01AH02	Alfentanil
N01AH03	Sufentanil
N01AH06	Remifentanil

**N01AX : Other general anaesthetics**	
N01AX03	Ketamine
N01AX07	Etomidate
N01AX11	Sodium oxybate
N01AX10	Propofol
N01AX13	Protoxyde d'azote
N01AX15	Xenon
N01AX	Flunitrazepam

N01B - LOCAL ANAESTHETICS

**N01BA : Esters of aminobenzoic acid**	
N01BA02	Procaïne
N01BA04	Chloroprocaïne

**N01BB : Amides**	
N01BB01	Bupivacaïne
N01BB03	Mepivacaïne
N01BB08	Articaïne
N01BB09	Ropivacaïne
N01BB10	Levobupivacaïne
N01BB51	Bupivacaïne associated with other drugs
N01BB53	Mepivacaïne associated with other drugs
N01BB58	Articaïne associated with other drugs

N02 - ANALGESICS

N02A - OPIOIDS

**N02AA : Natural alkaloids of opium**	

N02AA01	Morphine
N02AA03	Hydromorphone
N02AA05	Oxycodone
N02AA08	Dihydrocodeine
N02AA	Codeine

**N02AB : Phenylpiperidine derivatives**	
N02AB02	Pethidine
N02AB03	Fentanyl

**N02AC : Diphenylpropylamine derivatives**	
N02AC01	Dextromoramide

**N02AD : Benzomorphan derivatives**	
N02AD01	Pentazocine

**N02AE : Oripavine derivatives**	
N02AE01	Buprenorphine

**N02AF : Morphinan derivatives**	
N02AF02	Nalbuphine

**N02AX : Other opioids**	
N02AX02	Tramadol
N02AX06	Tapentadol
N03AX52	Tramadol associated with other drugs

N02B - OTHER ANALGESICS AND ANTIPYRETICS

**N02BG : Other analgesics and antipyretics**	
N02BG06	Nefopam
N02BG08	Ziconotide

N02C - MIGRAINE SUPPRESSANTS

**N02CC : Selective 5-HT1 receptor agonists**	
N02CC01	Sumatriptan
N02CC02	Naratriptan
N02CC03	Zolmitriptan
N02CC04	Rizatriptan
N02CC05	Almotriptan
N02CC06	Eletriptan
N02CC07	Frovatriptan

**N02CX : Other migraine suppressants**	
N02CX01	Pizotifen
N02CX06	Oxetorone
N02CX	Lysin acetylsalicylate + metoclopramide

N03 - ANTIEPILEPTICS

N03A - ANTIEPILEPTICS

**N03AA : Barbiturates and derivatives**	
N03AA02	Phenobarbital
N03AA03	Primidone
N03AA	Phenobarbital associated with other drugs

**N03AB : Hydantoin derivatives**	
N03AB02	Phenytoin
N03AB05	Fosphenytoin

**N03AD : Succinimide derivatives**	
N03AD01	Ethosuximide

**N03AE : Benzodiazepine derivatives**	
N03AE01	Clonazepam

**N03AF : Carboxamide derivatives**	
N03AF01	Carbamazepine
N03AF02	Oxcarbazepine
N03AF03	Rufinamide
N03AF04	Eslicarbazepine

**N03AG : Fatty acids derivatives**	
N03AG01	Valproic acid
N03AG02	Valpromide
N03AG04	Vigabatrin
N03AG05	Progabide
N03AG06	Tiagabine

**N03AX : Other antiepileptics**	
N03AX09	Lamotrigine
N03AX10	Felbamate
N03AX11	Topiramate
N03AX12	Gabapentin
N03AX14	Levetiracetam
N03AX15	Zonisamide
N03AX16	Pregabalin
N03AX17	Stiripentol
N03AX18	Lacosamide
N03AX21	Retigabine
N03AX22	Perampanel

N04 - ANTIPARKINSONIAN AGENTS

N04A - ANTICHOLINERGICS

**N04AA : Tertiary amines**	
N04AA01	Trihexyphenidyle
N04AA02	Biperidene
N04AA12	Tropatepine

N04B - DOPAMINERGICS

**N04BA : Dopa and derivatives**	
N04BA02	Levodopa + inhibitor of dopa-decarboxylase
N04BA03	Lévodopa + inhibitor of dopa-decarboxylase and catechol-O-methyltransferase

**N04BC : Dopaminergic agonists**	
N04BC01	Bromocriptine
N04BC02	Pergolide
N04BC04	Ropinirole

N04BC05	Pramipexole
N04BC06	Cabergoline
N04BC07	Apomorphine
N04BC08	Piribedil
N04BC09	Rotigotine
N04BC	Lisuride

**N04BX : Other dopaminergics**	
N04BX01	Tolcapone
N04BX02	Entacapone

N05 - PSYCHOLEPTICS

N05A - ANTIPSYCHOTICS

**N05AA : Phenothiazines with aliphatic chain**	
N05AA01	Chlorpromazine
N05AA02	Levomepromazine
N05AA06	Cyamemazine

**N05AB : Phenothiazines with piperazine structure**	
N05AB02	Fluphenazine
N05AB03	Perphenazine
N05AB06	Trifluoperazine
N05AB08	Thioproperazine

**N05AC : Phenothiazines with piperidine structure**	
N05AC01	Periciazine
N05AC02	Thioridazine
N05AC04	Pipotiazine

**N05AD : Butyrophenone derivatives**	
N05AD01	Haloperidol
N05AD05	Pipamperone
N05AD08	Droperidol
N05AD09	Fluanisone

**N05AE : Indole derivatives**	
N05AE03	Sertindole

**N05AF : Thioxanthene derivatives**	
N05AF01	Flupentixol
N05AF05	Zuclopenthixol

**N05AG : Diphenylbutylpiperidine derivatives**	
N05AG02	Pimozide
N05AG03	Penfluridol

**N05AH : Diazepines, oxazepines and thiazepines**	
N05AH01	Loxapine
N05AH02	Clozapine
N05AH03	Olanzapine
N05AH04	Quetiapine

**N05AL : Benzamides**	
N05AL01	Sulpiride
N05AL02	Sultopride

N05AL03	Tiapride
N05AL05	Amisulpride

**N05AN : Lithium**	
N05AN01	Lithium

**N05AX : Other antipsychotics**	
N05AX08	Risperidone
N05AX12	Aripiprazole
N05AX13	Paliperidone

N05B - ANXIOLYTICS

**N05BA : Benzodiazepine derivatives**	
N05BA01	Diazepam
N05BA02	Chlordiazepoxyde
N05BA03	Medazepam
N05BA04	Oxazepam
N05BA05	Clorazepate
N05BA06	Lorazepam
N05BA08	Bromazepam
N05BA09	Clobazam
N05BA011	Prazepam
N05BA012	Alprazolam
N05BA016	Nordazepam
N05BA018	Loflazepate
N05BA021	Clotiazepam
N05BA023	Tofisopam

**N05BB : Diphenylmethane derivatives**	
N05BB01	Hydroxyzine

N05C - HYPNOTICS AND SEDATIVES

**N05CA : Non associated barbiturates**	
N05CA03	Butobarbital

**N05CD : Benzodiazepine derivatives**	
N05CD01	Flurazepam
N05CD02	Nitrazepam
N05CD03	Flunitrazepam
N05CD04	Estazolam
N05CD05	Triazolam
N05CD06	Lormetazepam
N05CD07	Temazepam
N05CD08	Midazolam
N05CD09	Brotizolam
N05CD11	Loprazolam

**N05CF : Benzodiazepine-like agents**	
N05CF01	Zopiclone
N05CF02	Zolpidem

**N05CM : Other hypnotics and sedatives**	
N05CM16	Niaprazine
N05CM18	Dexmedetomidine

**N05CX : Hypnotics and sedatives in association**	

N05CX	Promethazine associated with other drugs

N06 - PSYCHOANALEPTICS

N06A - ANTIDEPRESSIVE AGENTS

**N06AA : Non-selective monoamine reuptake inhibitors**	
N06AA01	Desipramine
N06AA02	Imipramine
N06AA04	Clomipramine
N06AA05	Opipramol
N06AA06	Trimipramine
N06AA09	Amitriptyline
N06AA12	Doxepine
N06AA16	Dosulepine
N06AA17	Amoxapine
N06AA21	Maprotiline

**N06AB : Selective serotonin reuptake inhibitors**	
N06AB03	Fluoxetine
N06AB04	Citalopram
N06AB05	Paroxetine
N06AB06	Sertraline
N06AB08	Fluvoxamine
N06AB10	Escitalopram

**N06AF : Non-selective monoamine oxydase inhibitors**	
N06AF05	Iproniazide

**N06AG0 : Monoamine oxydase inhibitors (A type)**	
N06AG02	Moclobemide
N06AG03	Toloxatone

**N06AX : Other antidepressive agents**	
N06AX01	Oxitriptan
N06AX03	Mianserine
N06AX09	Viloxazine
N06AX11	Mirtazapine
N06AX12	Bupropion
N06AX14	Tianeptine
N06AX16	Venlafaxine
N06AX17	Milnacipran
N06AX21	Duloxetine
N06AX22	Agomelatine
N06AX26	Vortioxetine

N06B - PSYCHOSTIMULANTS, AGENTS USED IN ATTENTION DEFICIT HYPERACTIVITY DISORDER, AND NOOTROPICS

**N06BA : Centrally acting sympathomimetics**	
N06BA04	Methylphenidate
N06BA07	Modafinil

**N06BX : Other psychostimulants and nootropics**	
N06BX03	Piracetam

N06D - AGENTS USED IN DEMENTIA

**N06DA : Anticholinesterasics**	
N06DA02	Donepezil
N06DA03	Rivastigmine
N06DA04	Galantamine

**N06DX : Other agents used in dementia**	
N06DX01	Memantine

N07 - OTHER NERVOUS SYSTEM AGENTS

N07A - PARASYMPATHOMIMETICS

**N07AA : Anticholinesterase agents**	
N07AA01	Neostigmine
N07AA02	Pyridostigmine
N07AA30	Ambenomium

N07B - Agents used in substance dependence

**N07BB : Agents used in alcohol dependence**	
N07BB01	Disulfiram
N07BB04	Naltrexone
N07BB05	Nalmefene
N07BB	Disulfirame associated with other drugs

**N07BC : Agents used in opioid dependence**	
N07BC01	Buprenorphine
N07BC02	Methadone
N07BC51	Buprenorphine associated with other drugs

N07C - ANTI-VERTIGO AGENTS

**N07CA : Anti-vertigo agents**	
N07CA03	Flunarizine

N07X - OTHER NERVOUS SYSTEM AGENTS

**N07XX : Other nervous system agents**	
N07XX04	Sodium oxybate
N07XX05	Amifampridine
N07XX06	Tetrabenazine
N07XX07	Fampridine
N07XX11	Pitolisant

Reference

[1] Arrêté du 13 mars 2017 modifiant l'arrêté du 8 août 2008 pris pour l'application de l'article R. 5121-139 du code de la santé publique et relatif à l'apposition d'un pictogramme sur le conditionnement extérieur de certains médicaments et produit. Legifrance. 2017. https://www.legifrance.gouv.fr/eli/arrete/2017/3/13/AFSP1708232A/jo/texte. Published 18 March 2017.
