# Supplementary material for: Use of potentially driver-impairing drugs among older drivers
Source: BMC Geriatr. 2022 Jan 3;22:4. doi: 10.1186/s12877-021-02726-5 (PMC8722131; doi:10.1186/s12877-021-02726-5)
Supplement: Supplementary file 2 — Additional file 2. Number of PDI drugs categorized at level 2 or 3 in the French classification, recorded in the original study. Table summarizing the number of potentially driver-impairing drugs categorized at level 2 or 3 in the French classification, recorded in the original study. [file 12877_2021_2726_MOESM2_ESM.pdf]

Additional file 2. Number of PDI drugs categorized at level 2 or 3 in the French classification [1], recorded in the original study

	Number of distinct molecules	Number of prescriptions
**PDI drug level 2**	65	851
**PDI drug level 3**	12	545
**PDI drug level 2 or 3**	77	1396

Abbreviation. PDI, Potentially Driver-Impairing.

Reference

[1] Arrêté du 13 mars 2017 modifiant l'arrêté du 8 août 2008 pris pour l'application de l'article R. 5121-139 du code de la santé publique et relatif à l'apposition d'un pictogramme sur le conditionnement extérieur de certains médicaments et produit. Legifrance. 2017. https://www.legifrance.gouv.fr/eli/arrete/2017/3/13/AFSP1708232A/jo/texte. Published 18 March 2017.
