# Supplementary material for: Use of potentially driver-impairing drugs among older drivers
Source: BMC Geriatr. 2022 Jan 3;22:4. doi: 10.1186/s12877-021-02726-5 (PMC8722131; doi:10.1186/s12877-021-02726-5)
Supplement: Supplementary file 3 — Additional file 3. Multivariate analyses of factors associated with PDI-drug consumption in drivers, respectively in the 3 sub-cohorts. Table summarizing the multivariate analyses of factors associated with potentially driver-impairing drug consumption in drivers, respectively in the 3 sub-cohorts (chronic pain, atrial fibrillation and type 2 diabetes). [file 12877_2021_2726_MOESM3_ESM.pdf]

Additional file 3. Multivariate analyses of factors associated with PDI-drug consumption in drivers, respectively in the 3 sub-cohorts

	OR	95% CI	p-value
Chronic pain sub-cohort			
**ADL Score**	**0.26**	**0.11 – 0.56**	**0.001**
**Age**	1.00	0.96 – 1.00	0.724
**Female**	1.26	0.84 – 1.89	0.272
**History of depressive disorder**	**4.35**	**2.82 – 6.78**	**< 0.001**
Atrial fibrillation sub-cohort			
**Female**	0.86	0.46 – 1.55	0.627
**Age**	0.99	0.95 – 1.04	0.781
**Chronic pain**	**2.23**	**1.31 – 3.80**	**0.003**
**Polypharmacy**	**4.31**	**2.29 – 8.74**	**< 0.001**
**History of depressive disorder**	**7.52**	**4.11 – 13.85**	**< 0.001**
**Parkinson's disease**	**13.07**	**1.17 – 292.82**	**0.041**
Type 2 diabetes sub-cohort			
**Age**	0.98	0.94 – 1.03	0.549
**Female**	**1.94**	**1.10 – 3.41**	**0.022**
**Chronic pain**	**2.04**	**1.18 – 3.55**	**0.011**
**History of depressive disorder**	**3.85**	**2.12 – 6.95**	**< 0.001**
**Polypharmacy**	**3.61**	**1.76 – 8.24**	**< 0.001**

Abbreviations. PDI, Potentially Driver-Impairing; OR, Odds Ratio; 95% CI, 95% Confidence Interval; ADL score, Activities of Daily Living score (maximum score 6 indicating no disability in activities of daily living).
